# Managing Pain in Patients With Cancer: The Chinese Good Pain Management Experience

**DOI:** 10.1200/JGO.2016.005686

**Published:** 2016-09-21

**Authors:** Shi-Ying Yu, Jie-Jun Wang, Yu-guang Huang, Bing Hu, Kun Wang, Ping Ping Li, Yi-Long Wu, He-Long Zhang, Li Zhang, Qing-Yuan Zhang, Shu-Kui Qin

**Affiliations:** **Shi-Ying Yu**, Huazhong University of Science and Technology and Tongji Hospital, Wuhan; **Jie-Jun Wang**, Shanghai Changzheng Hospital, Shanghai; **Yu-guang Huang**, Peking Union Medical College Hospital; **Ping Ping Li**, Beijing University Cancer Hospital, Beijing; **Bing Hu**, Anhui Provincial Hospital, Anhui; **Kun Wang**, Tianjin Medical University Cancer Institute and Hospital, National Clinical Research Center for Cancer, Tianjin; **Yi-Long Wu**, Guangdong Lung Cancer Institute, Guangdong General Hospital, Guangdong; **He-Long Zhang**, Tangdu Hospital and Tangdu Comprehensive Cancer Center, Cancer Institute, 4th Military Medical University, Xi'an; **Li Zhang**, Sun Yat-Sen University Cancer Center, Guangzhou; **Qing-Yuan Zhang**, 3rd Affiliated Hospital of Harbin Medical University, Harbin, Heilongjiang; and **Shu-Kui Qin**, The People's Liberation Army Cancer Centre, Nanjing Bayi Hospital, Nanjing, Jiangsu, People’s Republic of China.

## Abstract

**Purpose:**

The number of cancer cases in China has increased rapidly from 2.1 million in 2000 to 4.3 million in 2015. As a consequence, pain management as an integral part of cancer treatment became an important health care issue. In March 2011, the Good Pain Management (GPM) program was launched to standardize the treatment of cancer pain and improve the quality of life for patients with cancer. With this work, we will describe the GPM program, its implementation experience, and highlight key lessons that can improve pain management for patients with cancer.

**Methods:**

We describe procedures for the selection, implementation, and assessment procedures for model cancer wards. We analyzed published results in areas of staff training and patient education, pain management in practice, analgesic drugs administration, and patient follow-up and satisfaction.

**Results:**

Pain management training enabled medical staff to accurately assess the level of pain and to provide effective pain relief through timely dispensation of medication. Patients with good knowledge of treatment of pain were able to overcome their aversion to opioid drugs and cooperate with nursing staff on pain assessment to achieve effective drug dose titration. Consumption of strong opioid drugs increased significantly; however, there was no change for weaker opioids. Higher pain remission rates were achieved for patients with moderate-to-severe pain levels. Proper patient follow-up after discharge enabled improved outcomes to be maintained.

**Conclusion:**

The GPM program has instituted a consistent and high standard of care for pain management at cancer wards and improved the quality of life for patients with cancer.

## INTRODUCTION

Pain is a common and persistent symptom in patients with cancer, occurring in 33% (95% CI, 21% to 46%) of patients who were cured of the disease and in the majority (64%; 95% CI, 58% to 69%) of patients with metastatic, advanced, or terminal disease.^1^ Pain intensity has also been shown to be positively correlated with depression,^2^ and longer pain duration and greater pain severity increase the risk of depression.^3^ Undertreatment of cancer pain is a widespread problem in Asia, where the percentage of patients with a negative pain management index (PMI), which represents the degree of undertreatment of pain, ranged from 27% to 79% (mean, 59.1 ± 17.5%).^4^ In comparison, the United States and Europe had lower mean negative PMIs of 39.1 ± 19.1% and 40.3 ± 26.6% respectively. In China, research on cancer registries estimated that there were 2.1 million cancer cases in 2000, but by 2012, the number of patients with cancer had increased by 45.6% to 3.1 million.^5,6^ By using high-quality data available from the National Central Cancer Registry of China, Chen et al^7^ estimated that the number of cancer cases in 2015 would increase rapidly to 4.3 million. As a consequence, pain management as an integral part of the treatment of cancer has become an important health care issue. Since 1990, Chinese health authorities have issued a number of guideline documents for the use of analgesic drugs for pain relief in the country; however, many patients continued to be inadequately treated even as pain medication and treatment protocols have become more widely available in recent years.^8^ A 2001 survey of 387 Chinese patients with cancer showed that 43% of them felt that they had been inadequately treated for their cancer pain.^9^ Another survey in 2009 of 531 patients who suffered from cancer pain at hospitals in Beijing showed that the situation has not improved significantly: 38% were not satisfied with the level of pain control received.^10^

In 1986, the WHO published the Cancer Pain Relief guidelines^11^ for the management of cancer-related pain. These guidelines recommended the careful assessment of the patient’s complaint of pain, together with his psychologic state, and use of alternative methods of pain control and a three-step analgesic-ladder approach for the prescription of pain relief drugs, starting from nonopioids (step 1), to weak opioids (step 2), and, finally, to strong opioids (step 3). The analgesic effect of nonopioids, such as nonsteroidal anti-inflammatory drugs, are dose dependent, that is, their effectiveness increases with increasing dosage; however, incidence of their adverse effects also increases at the same time.^12^ Opioids are also effective pain relievers and are indispensable for the treatment of moderate-to-severe cancer pain. Weak opioids, such as codeine and tramadol, are prescribed for patients with mild-to-moderate pain when nonopioid analgesics no longer provide adequate relief. Strong and high-potency opioids, such as morphine, oxycodone, hydromorphone, methadone, fentanyl, and buprenorphine, are used as a last resort for severe pain of which adequate relief with weak opioids is not achieved.^13^ However, physicians are often reluctant to use strong opioids for fear of their adverse effects, in particular, addiction.^14^ When used appropriately, consumption of opioids in a country can be a good proxy for determining the quality of palliative cancer care. Morphine-equivalence (ME) consumption and PMI, which are derived from WHO guidelines,^11^ are used to assess the efficacy of the treatment of cancer pain.^15^ In 1983, the per-capita consumption of strong opioids, excluding methadone, in China was low compared with the global average (0.33 ME *v* 2.22 ME). Although consumption increased to 0.75 ME in 2001, it remained low compared with the global rate (14.95 ME). However, the situation is improving: per-capita consumption in China rose to 2.95 ME in 2013, or a nine-fold increase over 30 years.^16^

The 2001 survey^9^ on cancer pain in China cited inadequate pain assessment, excessive state regulation on the prescription of opioids, inadequate staff knowledge of pain management, and lack of access to powerful analgesics as the main barriers to optimal management of cancer pain. In March 2011, the Ministry of Health of the People’s Republic of China launched the Good Pain Management (GPM) program to standardize the treatment of cancer pain, improve the quality of life for patients with cancer, and safeguard the quality and safety of health care services.^17^ The GPM program was initially targeted at oncology and terminal cancer treatment departments, pain specialist divisions, and palliative care wards at secondary and tertiary levels of general and cancer specialist hospitals. The program called for the creation of 150 such GPM model wards within a 3-year period as well as for playing a leading and exemplary role in improving and standardizing the quality of pain management.^8,17^ At the same time, standards for the selection and operation of the model wards, and importantly, the clinical management of pain were established to ensure treatment consistency and to raise the quality of pain management.^18,19^ This work describes the implementation of the GPM program, specifically its assessment and compliance processes at the model wards. We discuss some of the initial outcomes of the GPM program and highlight key lessons from its implementation, which we would like to share among physicians with a view toward improving pain management for patients with cancer in China and elsewhere.

## METHODS

Under the GPM program, the Ministry of Health Expert Group on the standardized treatment of cancer pain was formally established in March 2011 and was tasked with providing technical support and guidance for the creation of GPM wards and their related activities. Specific tasks included the development of cancer pain diagnosis and treatment guidelines; creation of standardized GPM demonstration wards; formulation of appraisal and auditing standards for GPM wards; preparation of training materials and organization of GPM-related training; provision of technical assistance to support the implementation of GPM wards; and, finally, analysis of outcomes and review of the GPM program.

The 41 members of the expert group comprised representatives from various disciplines involved in the management of cancer pain, including oncology, pain management, clinical pharmacy, nursing, hospice care, and opioids administration, and came from health care institutions at different geographical regions across the country. There were 26 oncologists, nine pain management specialists, three clinical pharmacists, and one specialist each from nursing, hospice care, and opioids administration. These members came from 19 cities and represented 33 hospitals and medical institutions across China.

### Selection of GPM Wards

**Fig 1:**
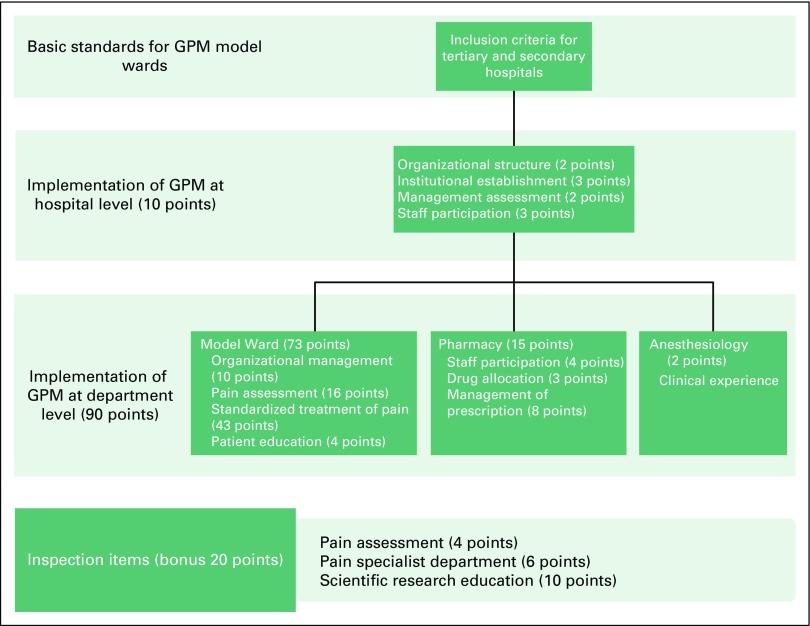
Structure of the Good Pain Management (GPM) program assessment checklist.

We developed a standard GPM assessment checklist in accordance with GPM guidelines^20^ to assist health administrators in evaluating and nominating suitable cancer treatment wards for the GPM program; to guide these selected model wards in the implementation of GPM; and to audit the model ward implementation for compliance with GPM guidelines.^17^ Figure 1 describes the structure of this GPM assessment checklist. Model wards for the GPM program were selected from oncology wards at cancer specialist hospitals and pain specialist departments at the secondary and tertiary levels of the health care system. Inclusion criteria were based on the duration of clinical practice, number of beds and admissions, annual number of cases, and staff training. Oncology wards were additionally assessed on their technical competence and ability to setup an independent outpatient pain management service for their patients (Table 1).^19^

**Table 1:**
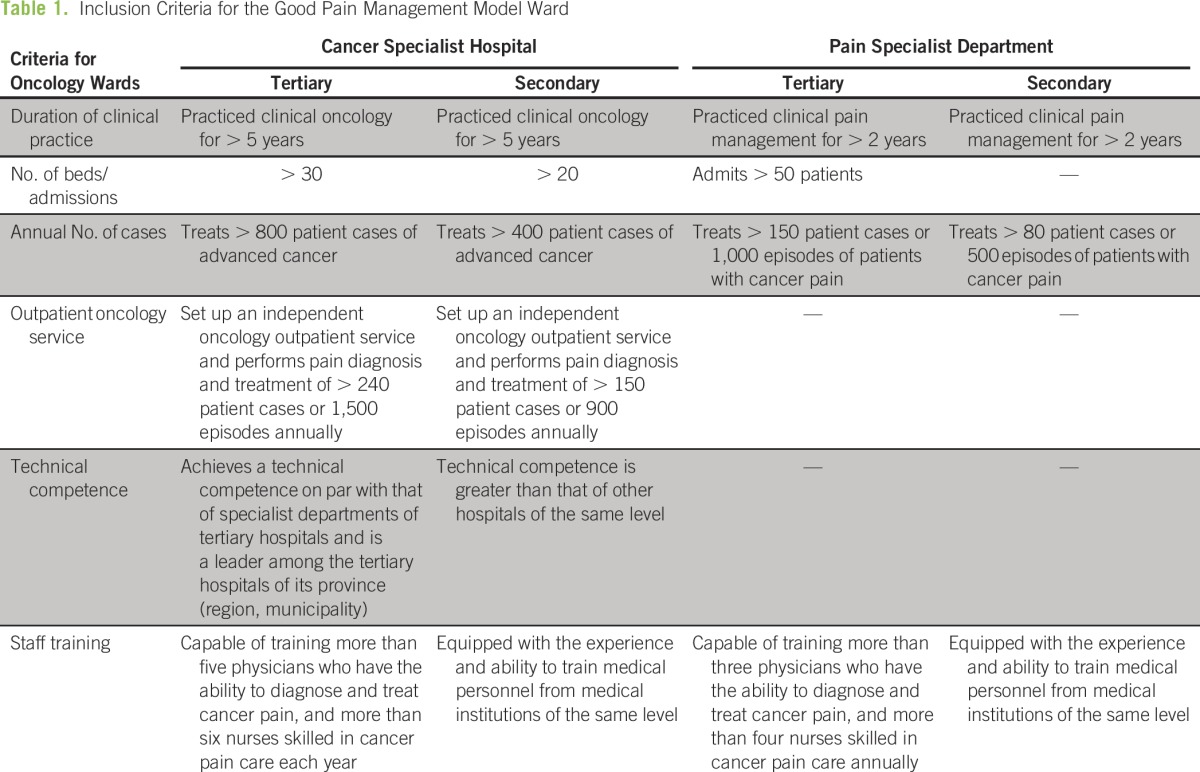
Inclusion Criteria for the Good Pain Management Model Ward

At each nominated GPM hospital, a model ward project panel was established to plan, oversee, and coordinate its implementation among the affected departments, namely, the oncology ward (the model ward), the pharmacy, and anesthesiology, as well as to ensure compliance through regular audits. A project team then developed the requisite protocols and carried out the implementation of the GPM program at these departments. A medical affairs department was also created within the hospital and was responsible for the review of pain education activities for health care professionals and patients and their families to identify areas of concern for rectification. These reviews also focused on the effectiveness of pain treatment, quality of medical record-keeping, analyses of cause of death, and patient quality of life and post-treatment follow-up.

External audit teams at provincial and national levels conducted audits for the certification of model wards. Each audit team was composed of an oncologist, a pain management specialist, a nurse, a pharmacist, and an audit coordinator who were selected from certified GPM wards. The team performed an independent audit of the implementation for compliance by using the GPM assessment checklist. This on-site audit, which covered the model ward, outpatient pain clinic, pharmacy, and hospital administration department, was conducted through interviews of patients and staff and inspection of medical documentation. The audit team highlighted the shortfalls in implementation and recommended remedial courses of action. Further audits were conducted annually at the provincial level to ensure that certified wards maintained their GPM standards.

### GPM Assessment Checklist

The GPM assessment checklist used a point system to guide the systematic implementation of the program, with more points awarded to areas of greater emphasis. A maximum of 100 points—10 points at the hospital level and 90 points at the department level—were awarded, with an additional 20 bonus points given for additional capabilities, such as pain management expertise, staff training, and medical research (Fig 1). In the assessment of model ward performance, which carried the highest number of points (73 points), emphasis was placed on pain assessment (16 points) and the standardized treatment of pain (43 points). Staff participation, including training and patient education, was also emphasized at hospital and departmental levels.

### Pain Assessment

The practice of cancer pain management, that is, pain diagnosis, treatment processes, and use of analgesic drugs, was standardized under the Standardized Diagnosis and Treatment Protocol for Cancer Pain (2011 Edition) document^18^ to ensure consistency in the classification of pain and assessment and treatment methodologies for patients with cancer. This document recommends that an overall patient pain assessment be completed within 8 hours upon admission and that regular pain assessments using the brief pain inventory^18^ be included as part of the nursing routine and carried out at regular intervals during patient stays. This pain assessment used a dynamic evaluation mechanism that measured the pain level, changes in the nature of pain, acute pain episodes, determinants of pain relief, and aggravation and adverse reactions to medication.

Quantitative pain assessment was performed by using the numerical rating scale. The numerical rating scale has a 0- to 10-point scale, where 0 equals no pain and 10 equals maximum pain. The verbal rating scale (VRS), simple pain assessment scale, or the visual analog scale was used for patients who had difficulty communicating their pain level, such as children, the elderly, or patients with communication difficulties, such as language or cultural differences. The VRS has a 4-point Likert-like scale: no pain, mild pain, moderate pain, and severe pain. These numerical and verbal scales were often used interchangeably, and the following equivalence was applied: 0 = no pain, 1 to 3 = mild pain, 4 to 6 = moderate pain, and 7 to 10 = severe pain. Improvement in pain relief was described by using a 5-point scale—no relief, mild relief, moderate relief, apparent relief, and complete relief.

### Standardized Treatment of Pain

A system of patient informed consent was established, and patients and their families were informed of the purpose of pain management and its attendant risks, precautions, and possible adverse reactions before treatment of cancer pain. The prescription of analgesic therapy was based on the WHO three-step analgesic ladder^11^ and was administered at regular intervals rather than on demand, with oral administration preferred over the transdermal route. Treatment was adjusted according to the patient’s changing pain condition. This personalized, case-by-case treatment plan for which treatment is based on patient condition and physical health—and was developed collectively by oncology, pain management, and pharmacy departments—was targeted to achieve a treatment efficiency of ≥ 75%.^19^

Consumption of opioids (steps 2 and 3) drugs at hospitals was measured by the daily defined dose^21^ or by their unit of prescription, for example, tablet, injection, or suppository. The combined use of opioids with nonsteroidal anti-inflammatory drugs was encouraged to enhance the analgesic effects and reduce opioid consumption. A patient who used opioids for the first time was given short-acting agents, for example, immediate-release morphine tablets. When long-acting opioids, such as sustained-release morphine or oxycodone tablets, were used, short-acting opioid analgesics were prepared as a rescue medication. Whereas adverse reactions to opioid drugs were mostly temporary or tolerable, the prevention and treatment of such reactions formed an important part of the pain treatment plan, and patients who experienced adverse effects were monitored for reduced renal function, hypercalcemia, metabolic abnormalities, and combination use of psychotropic drugs. Rescue medication for opioid-associated adverse events was also made available. In the event of excessive sedation or mental health disorders, the dosage of analgesic drugs was reduced.

### Training and Education

The GPM program called for the establishment of a medical training system that would ensure that all cancer-related health care professionals received training in pain management at least once a year. Effectiveness of the training was assessed by testing their knowledge of pain assessment and management. Patients and their families were also provided information on cancer pain management through publicity and education seminars that were conducted on a regular basis as well as through education billboards. Patient knowledge of pain management was assessed through surveys.

### Patient Follow-Up After Discharge

A follow-up system for discharged patients was established, with a targeted follow-up rate of ≥ 70%.^19^ Follow-up by telephone was performed within 1 week of discharge. Regular visits after discharge to conduct pain assessment were carried out to ensure that patients received sustained and effective treatment.

We hand-curated publicly available reports on the GPM program in medical journals and evaluated its implementation outcomes in the following areas: training and education of medical staff and patients; good pain management in daily practice; analgesic drug administration; and patient follow-up after discharge and evaluation.^22^ Where the data were available, the significance of these outcomes was evaluated at the *P* = .05 level.

## RESULTS

Of 150 GPM model wards to be established within 3 years of program inception, 100 were to be located at tertiary hospitals and 50 at secondary hospitals. At the end of 2012, 66 such wards successfully completed a series of assessments and were certified as GPM model wards.^8^

### Training and Education of Medical Staff and Patients

Table 2 shows the observed outcomes of GPM training programs at four hospitals, measured through assessment of medical staff and patients on their knowledge of good pain management. Test scores for physicians and nursing staff on pain management and assessment knowledge were found to be significantly higher after the staff had completed their training (*P* < .05). In addition, nursing staff at the Shenzhen Nanshan People’s Hospital also reported a greater sense of professional accomplishment after their training.^23^

**Table 2:**
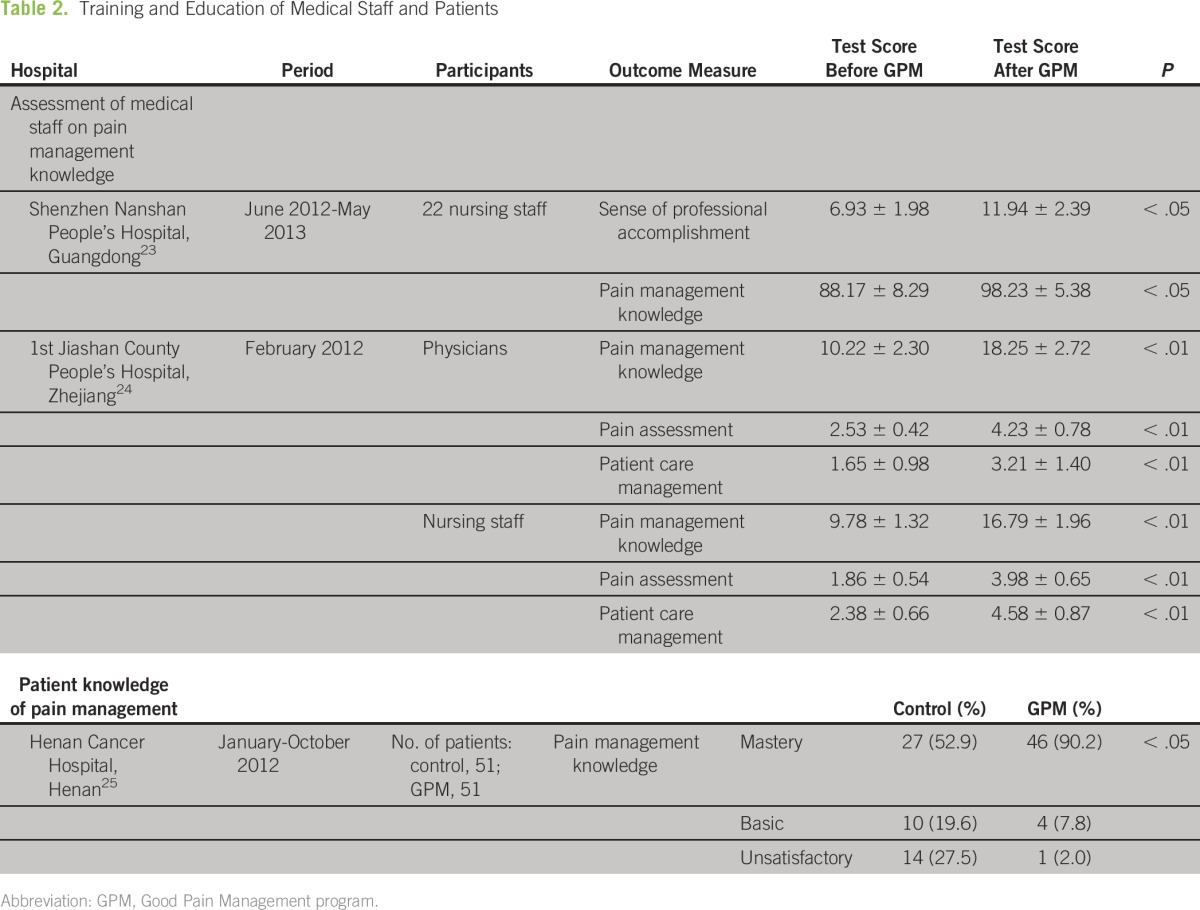
Training and Education of Medical Staff and Patients

Henan Cancer Hospital instituted a health education program for all admitted patients that covered knowledge of pain and its treatment, treatment and care routines for pain, and nonpharmacologic methods of pain relief and guidance on self-care postdischarge.^25^ Nursing staff evaluated two groups of patients, GPM and control, on their knowledge of pain management at three levels: mastery, basic, or unsatisfactory. The difference in mastery levels between GPM and control groups was significant (*P* < .05).

### Good Pain Management in Daily Practice

Table 3 shows the impact of the GPM program on pain management before and after its implementation at four hospitals. VRS was the most commonly used method of pain assessment. After 2 weeks of treatment, remission rates for patients with moderate and severe pain at Tongji Hospital Cancer Centre were 24.3% ([189 − 143 = 46] of 189) and 38.3% ([47 − 29 = 18] of 47), respectively. These rates improved after a further 2 weeks to 72.0% ([189 − 53 = 136] of 189) and 95.7% ([47 − 2 = 45] of 47), respectively. At Sun Yat-sen University Cancer Centre, patients were divided into control and GPM groups. Complete (no pain) remission rate for the GPM group was significantly higher than that for the control group (54.5% [79 of 145] *v* 33.7% [31 of 92]; *P* < .05). Similarly, for patients with moderate or severe pain, the remission rate was significantly higher for the GPM group (decreased to mild or none; 82.6% [81 of 98] *v* 62.3% [48 of 77]; *P* < .05).^22^ The 1st Affiliated Hospital of Dalian Medical University used brief pain inventory scores to record the pre- and post-GPM at the most severe, least severe, average, and current pain levels in the previous 24 hours. Patients reported significant improvements in pain relief at all pain levels after GPM was adopted (*P* < .05).^27^ The Beijing Chest Hospital, which established a GPM pain clinic for outpatients in April 2012, assessed that 73.1% of its outpatients during the next 2 months (April to June 2012) had moderate-to-severe pain before treatment on a daily basis.^28^ After GPM treatment, this percentage dropped to 5.8% and approximately 65.2% of patients reported no pain.

**Table 3:**
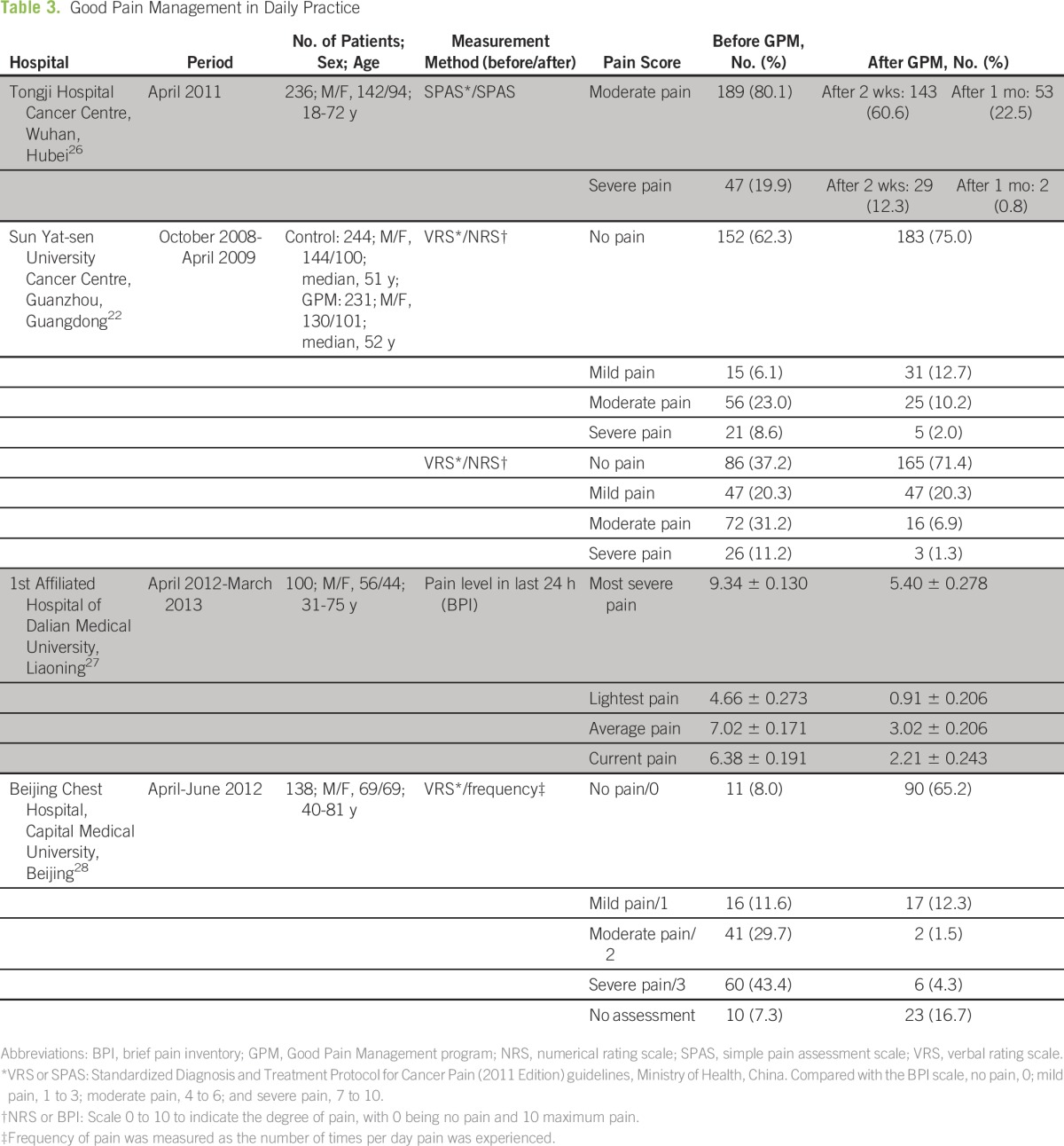
Good Pain Management in Daily Practice

### Analgesic Drug Administration

Table 4 shows the consumption of strong (step 3) and weak (step 2) opioids before and after implementation of GPM at three hospitals. Morphine and oxycodone were the two most commonly prescribed strong opioids. Morphine sulfate (sustained-release tablets) was the most-used opioid at the Ganzhou and Hubei Cancer Hospitals and the second most-used opioid at the 2nd Affiliated Hospital of Zhejiang University. In addition, there was an increase in the postimplementation use of morphine sulfate, which ranged from 23.8% to 51.1%. Oxycodone (sustained-release tablets) was most consumed at the Zhejiang University Hospital and saw a three-fold (287.8%) increase in use post-GPM implementation. Consumption of weak opioids at the Ganzhou and Hubei Cancer Hospitals mostly decreased or was little changed after GPM implementation.

**Table 4:**
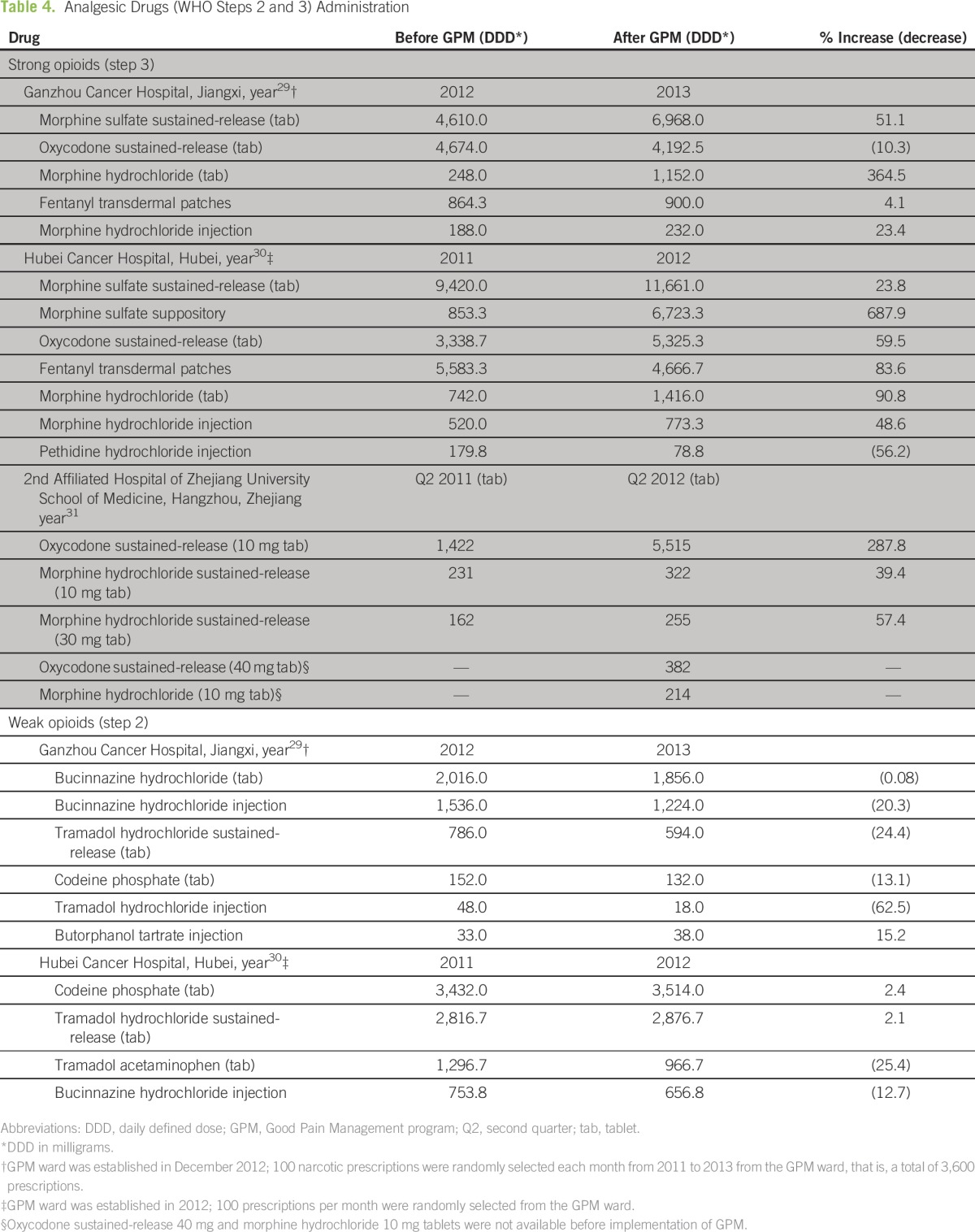
Analgesic Drugs (WHO Steps 2 and 3) Administration

### Patient Follow-Up After Discharge and Evaluation

Ninghe County Hospital and the 1st People’s Hospital of Jingzhou in Hubei applied guideline-based pain treatment in at least eight in 10 patients after GPM implementation (Table 5). An analysis of 74 patients at the Ninghe County Hospital found that only 42 of patients (57%) had received guideline-based pain treatment before GPM implementation. Of the noncompliant cases, 16 involved irregularities in treatment process that were avoidable and three had errors in pain assessment. Remedial efforts included training seminars for medical staff on pain management guidelines and management of narcotic drug and adverse drug reaction. Pain assessment procedures were standardized by using the visual analog scale, and physician and nursing staff were required to conduct the assessment together.^32^ This resulted in an improved GPM-compliant treatment rate of 84% at 6 months after implementation.

**Table 5:**
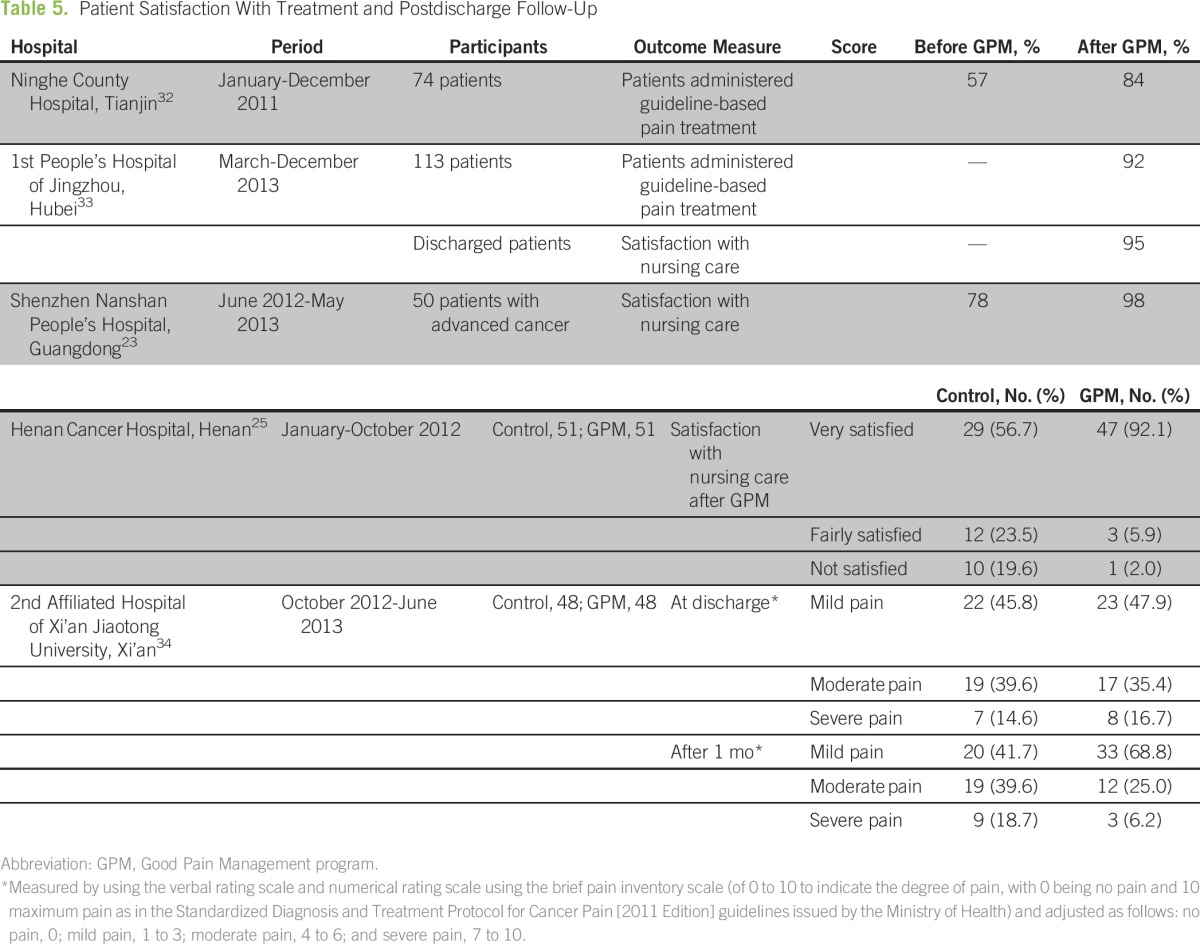
Patient Satisfaction With Treatment and Postdischarge Follow-Up

Surveys of the patient satisfaction with nursing care outcome at model wards in the Jingzhou, Shenzhen, and Henan hospitals showed that more than nine in 10 patients were satisfied with the treatment they received (Table 5). At the Henan Cancer Hospital, there was a difference between the satisfaction rates of control and GPM groups after the GPM program was implemented (*P* < .05).^25^

The 2nd Affiliated Hospital of Xi’an observed discharged patients at discharge and 1 month after discharge. Although pain profiles of control and GPM groups were similar at discharge, there was a difference in the pain profile of the GPM group at 1 month after discharge compared with control group (*P* < .05): the GPM group had achieved noticeably better pain management outcomes.^34^

## DISCUSSION

Our study of the published results of GPM implementation covered approximately 19.7% (13 hospitals) of the 66 certified model wards under the first phase of the GPM program. We are aware that these results are limited and may not be fully representative of the current state of the program. However, the outcomes and patient experiences at these model wards, which are located in nine (26.5%) of the 34 provinces and municipalities in China, can provide an overview of the implementation of the GPM program across the country and provide useful learning points for pain practitioners who want to improve the level of care given to patients with cancer.

According to a face-to-face survey of 500 Chinese physicians who treated patients with cancer at 11 general hospitals in Sichuan, China, between December 2011 and Dececember 2013, the main barriers to better pain management were reported as inadequate medical knowledge, pain assessment and its management, and patient reluctance to use opioids for fear of addiction, drug tolerance, and adverse effects.^29^ Compared with with the earlier 2001 survey,^9^ excessive state regulation on the prescription of opioids and lack of access to powerful analgesics were no longer reported as barriers; however, inadequate pain assessment and staff knowledge of pain management remained as barriers to pain management. Our analysis of the available data on GPM model wards showed that, when given appropriate training in pain management standards and procedures, medical staff were able to accurately assess the level of pain of patients and to provide effective pain relief through correct and timely dispensation of pain medication. Appropriate training also gave nursing staff a greater sense of professional accomplishment, raised their awareness, and increased their learning motivation toward good pain management.^33^ The GPM program also saw an increased use of strong opioids, which led to higher pain remission rates, especially for patients with moderate-to-severe pain. Improved patient education at model wards also helped patients overcome their aversion to these drugs and increased their willingness to report pain symptoms.^22^ We also noticed that use of weak opioids decreased, although the underlying reasons may require further analysis. Within the GPM program, proper pain and drug use record-keeping were instituted at the model wards and this reduced the number of instances of inappropriate use of analgesic drugs.^29-31^ Good record-keeping helped provide relevant information on drug prescriptions for patients upon discharge. As the hospitals that implemented GPM continued to support patients after discharge, good record-keeping also helped to support patient follow-up monitoring and postdischarge treatment and enabled improved patient outcomes to be maintained for longer periods.^34,36^

An on-site assessment of GPM model wards at 30 hospitals in the Zhejiang Province, China, was conducted in June and July 2012, the results of which lend credence to our views.^37^ Assessors found that nursing staff had conducted pain assessment in a timely manner and that patients who had a good knowledge of pain treatment were able to cooperate with the nursing staff on their pain assessment and drug dose titration. The ≥ 70% target rate for telephone follow-up after discharge was achieved by all but one hospital. The study also highlighted several areas for improvement: pain assessment was conducted too frequently, especially during the night, and may have affected the patient’s rest; the observational skills of nursing staff could be improved in such areas as visual (facial) pain assessment and adverse drug reaction; a need for pain assessment—in addition to the location of the pain and its intensity—must address the psychologic, emotional, social, and cultural aspects; and better storage and management of pain medication to ensure that it is available for timely dispensation when needed.

As each hospital conducted its own independent study of its GPM program, measured outcomes from one hospital could not be directly compared with those from other hospitals. Past records of pain parameters, such as pain score, opioid drug dose titration, dynamic changes in pain, and adverse reactions, may be incomplete as pain is one of the many symptoms of cancer. In addition, there was no uniform scale for pain measurement used across the hospitals. Whereas this may place some limitations on the interpretation of the success of the GPM program, we believe that the program delivered concrete and practical guidelines which have enhanced the diagnosis and treatment of cancer pain and, at the same time, improved the clinical management processes at these hospitals. This has encouraged the adoption of GPM practices at health care institutions in China.

By early 2016, 67 national wards and 769 provincial wards have been certified, as more hospitals implemented and adhered to the common set of guidelines for the establishment of GPM wards, procedures for pain assessment, and standards for pain treatment and management. This brings the total to 836 model wards, which far exceeds the 150 model wards initially planned for the GPM program—a testament to its success. The consistent and visible improvements to patient care brought about by the GPM program at the model wards has provided a useful benchmark for the degree of improvement that can be achieved in real-life practice.
